# Tuning of Elasticsearch Configuration: Parameter Optimization Through Simultaneous Perturbation Stochastic Approximation

**DOI:** 10.3389/fdata.2022.686416

**Published:** 2022-05-11

**Authors:** Hårek Haugerud, Mohamad Sobhie, Anis Yazidi

**Affiliations:** ^1^Department of Computer Science, OsloMet—Oslo Metropolitan University, Oslo, Norway; ^2^Department of Informatics, University of Oslo, Oslo, Norway

**Keywords:** Elasticsearch, configuration, Simultaneous Perturbation Stochastic Approximation, parameter tuning, optimization

## Abstract

Elasticsearch is currently the most popular search engine for full-text database management systems. By default, its configuration does not change while it receives data. However, when Elasticsearch stores a large amount of data over time, the default configuration becomes an obstacle to improving performance. In addition, the servers that host Elasticsearch may have limited resources, such as internal memory and CPU. A general solution to these problems is to dynamically tune the configuration parameters of Elasticsearch in order to improve its performance. The sheer number of parameters involved in this configuration makes it a complex task. In this work, we apply the Simultaneous Perturbation Stochastic Approximation method for optimizing Elasticsearch with multiple unknown parameters. Using this algorithm, our implementation optimizes the Elasticsearch configuration parameters by observing the performance and automatically changing the configuration to improve performance. The proposed solution makes it possible to change the configuration parameters of Elasticsearch automatically without having to restart the currently running instance of Elasticsearch. The results show a higher than 40% improvement in the combined data insertion capacity and the system's response time.

## 1. Introduction

The amount of data generated every day is increasing at a remarkable pace and, as of 2017, 3.8 billion people are using the internet. It was estimated that 1.7 MB of data will be created for every person every second in 2020 (Domo, 2018). A particular kind of data is generated by servers to maintain their status. These data are stored in files, often referred to as log files. They can include different types of data, such as web requests from users, user activities, server events, etc. Log files are considered a part of big data (Jacobs, 2009; Oussous et al., 2018). Due to the increase in the number of users and machines, there are enormous amounts of data to analyze.

Different aspects are important when discussing big data, one of which is searching such data. When it comes to search methods, the time it takes to retrieve the right information means that the quality and speed of the search engine is crucial. The existence of big data has led to a need for good search engines in which information can be quickly collected that is relevant to the input searched for. Elasticsearch is a search engine that became popular within the Development and Operations (DevOps) field, and also among many tech companies. It can be combined with other tools that collect logs from servers and visualize them. Elasticsearch technology is used by large companies like eBay, Uber, and Netflix and the company behind this technology, Elastic, was named a visionary in a 2021 Gartner report on application performance monitoring (De Silva and Padraig Byrne, 2021).

The traditional way of operating development teams in organizations has led to less efficiency and more conflicts when new features or updates are pushed to production (König and Steffens, 2018). There is therefore an urgent need to fill the gap between development and operations, which has resulted in the creation of the DevOps field, that is, a field in which operations, development, and quality assurance teams are unified (Ebert et al., 2016). This has made it possible to release new code into production faster, and it has also increased the quality of software systems (Bezemer et al., 2018).

Even though the current DevOps tools largely do not rely on machine learning (ML), there has been some interest in applying machine learning in DevOps tools where the quality of software processes has been enhanced (Nogueira et al., 2018). This has led to attention being devoted to how to increase quality by applying optimization solutions. Elasticsearch configuration relies on a large number of parameters, which means that tuning the right parameters will yield better results in terms of resource use and fast output, and hence quality. An example of a substantial Elasticsearch deployment is the one at CERN with more that 30 Elaticsearch clusters and four hundred nodes. One of the lessons learned at CERN was that the behavior of clusters can change drastically depending on the user patterns, and that this should be closely monitored (Saiz and Schwickerath, 2020). The many installations of Elastisearch worldwide, the large configuration space, and the dynamic behavior of the user patterns motivate the goal of this article, an automated and dynamic parameter tuning of Elasticsearch in order to improve the efficiency of the data insertion capacity and the response time of the system. Our approach is to combine machine learning and Elasticsearch, resulting in a dynamic algorithm which improves the performance of Elasticsearch by tuning some of its configuration parameters.

## 2. Related Work

Tuning the configuration to achieve better performance has always been a practice among researchers and system administrators. Big data processing systems such as Hadoop, Spark, and Storm all have a large number of configuration parameters that must be tuned in order to optimize performance and stability. Because of the vast number of parameters, it is not feasible to obtain an optimal configuration manually and there is a large body of research on automated parameter tuning (Herodotou et al., 2020). A recent auto-tuning trend involves using machine learning in order to find the optimal configuration of a system (Zhou et al., 2022). One such approach consists of performance tuning Apache Drill on Hadoop clusters using evolutionary algorithms (Bløtekjær, 2018). The solution automates the process of fine-tuning the Drill configuration, and experimental results show that the performance of Drill is boosted compared to the default configuration. A similar approach uses a genetic algorithm to solve high-dimensional challenges in Hadoop (Yildirim et al., 2015). The solution consists of using a large population and then evolving it through cycles of a genetic algorithm. Another algorithm that has been used to tune the parameters of a Hadoop system is the SPSA (Simultaneous Perturbation Stochastic Approximation) algorithm from Kumar et al. (2016). This work shows the effectiveness of using two system observations per iteration in order to tune the configuration parameters. A self-tuning approach for Apache Spark based on an artificial neural network was proposed by Rahman et al. (2018).

It is worth mentioning that Elasticsearch is a NoSQL database and that there has been a fair amount of research on optimizing NoSQL databases, such as MongoDB, Cassandra, CouchD, Riak, HBase, and Redis.

An automatic configuration-tuning framework for NoSQL database benchmarking, *ConfAdvisor*, treats database performance as a black-box function of its configuration parameters (Chen et al., 2020). Various black-box optimization algorithms are used, including Bayesian optimization, and the experiments show a substantial improvement in performance.

A model agnostic optimization framework for configuration in the cloud, called Morphling, was recently proposed (Wang et al., 2021). The model tackles the high dimensionality problem that Bayesian optimization approaches face by deploying a meta-learning approach that helps reduce the sampling cost. The meta-learning approach makes it possible to predict the performance trends under varying workloads and thereby to reduce the search by proposing a small set of configurations to sample from.

A similar approach to the one presented here uses genetic algorithms to evolve the configuration of Elasticsearch (Lu et al., 2020). Machine learning algorithms, namely random forest and gradient boosting regression trees, were used to predict the performance of the configuration.

The optimization of the Elasticsearch search engine is a related research field (Coviaux, 2019). In this case, there are also a large number of configuration parameters, and an experimental setup for automating the configuration of Elasticsearch was devised in Silva-Muñoz et al. (2021). Bayesian optimization cannot be directly utilized to solve such a high dimensional black-box optimization problem due to the curse of dimensionality. In this perspective, Silva et al. resorted to the iRace optimization package (López-Ibáñez et al., 2016) in order to auto-tune the parameters of two NoSQL databases, namely Cassandra and Elasticsearch.

In Dou et al. (2020), the authors present *HDConfigor*, an automatic full-stack configuration parameter tuning tool for log search engines. *HDConfigor* solves the high dimensional black-box optimization problem by using an algorithm that introduces a random embedding matrix to generate an embedded space. It then performs Bayesian optimization in this low dimensional embedded space.

In Mahgoub et al. (2020), the authors tackle the problem of optimizing the configuration of an NoSQL database in the cloud with the aim of minimizing the performance per dollar under a given budget. The optimization problem is NP-hard. Indeed, if we consider a cluster of *N* nodes, and *I* VM configurations for each node, the search space is in the order of *I*^*N*^. The authors propose a search algorithm called OPTIMUSCLOUD, which aspires to partition the cluster into so-called Complete-Sets. A complete-set is defined as the minimum number of nodes that have records that cover all the records in the database. Since the performance of a complete-set is limited by the performance of the slowest server, it is suggested that all nodes in the complete-set should be reconfigured to match the fastest node speed. In a similar manner to Lu et al. (2020), a random forest is trained to predict the performance of the NoSQL server for different configurations of VMs and databases.

{SOPHIA} (Mahgoub et al., 2019) present another thread of approaches for optimizing online, i.e., during runtime, the configuration of NoSQL databases under a time-varying load. Please note that such optimization is different from most approaches, which are offline. The intuition behind {SOPHIA} is to monitor the performance of the NoSQL database and perform a cost benefit analysis to assess the effect of the configuration switch. A genetic algorithm is used to find a configuration plan. Similarly, when it comes to online configuration of NoSQL databases, Preuveneers and Joosen (2020) use adaptive Hoeffding tree as a machine-learning algorithm to predict the best configuration online. The authors employ middleware to monitor the performance, detect performance degradation, and then predict the best configuration based on the machine-learning algorithm.

The informed reader will note that all the aforementioned works employ some sort of search algorithm to explore the configuration space, such as genetic algorithms or Bayesian optimization. In addition, some of the above approaches use supervised learning to predict the performance of a certain configuration based on historic data. Interestingly, due to the increasing development of machine learning, some recent trends have emerged, such as the use of deep reinforcement learning for auto-configuration of Apache Storm during runtime (Li et al., 2018). Another important and emerging trend in auto-configuration relies on the use of generative adversarial networks (GANs) to generate robust configurations (Bao et al., 2019). The underlying idea of ACTGAN (Bao et al., 2019) is that “good” configurations share some underlying common hidden patterns, which a GAN can unveil. Experiments using different software traces including NoSQL databases, such as Redis, Cassandra, and HBase, demonstrate the effectiveness of a GAN when applied to auto-configuration.

## 3. Experimental Setup

### 3.1. Objectives

The objective of this project is to optimize the configuration of Elasticsearch using an optimization algorithm that is based on simultaneous perturbation. The algorithm is implemented on an Elasticsearch container cluster. The goal is to be able to tune the configuration of the cluster by running benchmarks and analyzing them through the algorithm, and then enhancing the configuration on each iteration. For each iteration, the indexing metric and response time are the factors that influence the optimization process. The benchmark process examines the performance of the executing Elasticsearch node by inserting data into it and removing the data once the analysis is finished. ESRally is an open-source tool that is used to benchmark Elasticsearch and is available on Github (Mitterdorfer, 2022a). The source code of the experimental setup is also available on Github (Sobhie, 2022).

### 3.2. Elasticsearch Metrics

There are several factors that play a crucial part in the performance of Elasticsearch. The metrics are context-dependent, however, and since different systems can run on top of Elasticsearch (Bai, 2013), more metrics will be considered as well. The following are metrics to consider in Elasticsearch (Subhani Shaik, 2017).

When adding an index to a node, it is necessary to use shards. The index is subdivided into multiple pieces, and these new pieces are called shards (Kuc and Rogozinski, 2013). Cluster status shows information inside the cluster components, such as running nodes and how many shards are assigned. It also provides information about the time it takes a cluster to allocate shards.

The node performance is dependent on the specifications of the machine in which the node is installed. Assets like the CPU, RAM, and operating system will affect performance. There are a few parameters that help to optimize and assess index performance. Indexing latency can be calculated using the available parameters **index_total** and **index_time_in_millis**. Another metric is the Flush latency that helps to detect problems with disks. When there is a problem with slow disks, this flush latency metric will increase. Querying is used when making search requests. The number of queries written and how they are written will influence the performance of a node. Because of that, Query Latency and Query Load are two important metrics to monitor.

**Figure 1:**
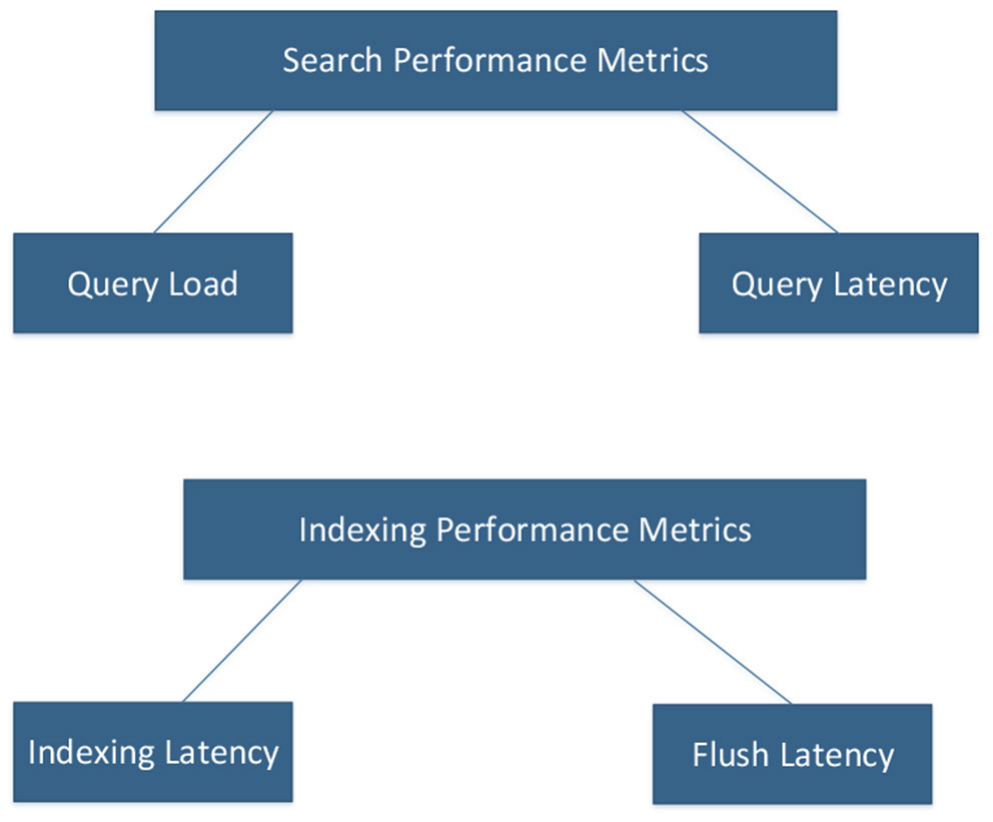
Elasticsearch performance metrics.

Both Index and Search performance metrics can be summarized as shown in Figure 1. Query Load and Query Latency influence the performance of searching, while Index Latency and Flush Latency affect Indexing Performance.

Writing a proper search query is the main factor influencing search performance in Elasticsearch. Similarly, factors like data type and how they are organized also play a role. However, to increase the speed of the search, there are two important methods that can be used (Subhani Shaik, 2017), custom routing, and force merging which will be explained in the next two paragraphs.

When there are several shards in a node, Elasticsearch checks all segments inside each shard, not all shards, just the ones that satisfy the search request. Custom routing makes it possible to store the chosen data on the same shard. Only one shard will thereby be searched in order to satisfy the query. As a result, fewer shards need to be investigated. Similarly, it is possible to decrease the number of segments in each shard by using the Force Merge API.

The purpose of Force Merge is to merge segments continuously until the value of **max_num_segments** in a shard is reduced to 1. However, when the number of segments and shards is high, the force merging process will be slow. For example, merging 10,000 segments to 5,000 segments takes less time than merging 10,000 segments to one. This will affect the resources required to perform the process, which will also affect the search requests. In that case, it is recommended to schedule Force Merging during non-busy hours.

There are many parameters to consider as regards both searching and indexing speed in Elasticsearch. Table 1 summarizes most of the parameters that have an influence on indexing performance, and hence search performance.

**Table 1:** Elasticsearch tuning parameters.

**Parameter**	**Description**
index.refresh.interval	Time to wait before copying in-buffer memory
index.number.of.replicas	The number of replicas each primary shard has
indices.memory.index.buffer.size	Allocation of heap memory
indices.memory.min.index.buffer.size	Allocation of heap memory
indices.memory.max.index.buffer.size	Allocation of heap memory
index.translog.flush.threshold.size	Make a flush after reaching specific size
index.translog.retention.age	Duration for keeping a translog file
index.translog.sync.interval	How often the translog is synced to disk
index.number.of.shards	The number of primary shards per index
index.shard.check.on.startup	Shards should be checked for corruption before opening

### 3.3. Infrastructure Overview

**Figure 2:**
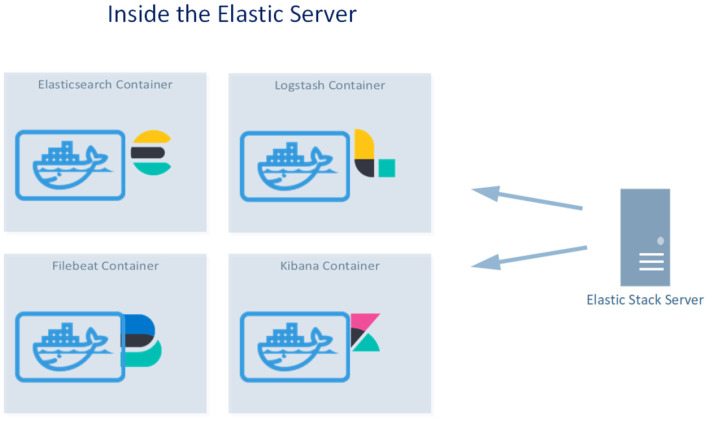
The four docker containers running inside the Elastic Stack Server.

The infrastructure of the project consists of a server hosting the Elastic Stack applications and several virtual machines that send their logs to the Elastic cluster. All the applications run in docker containers. Figure 2 shows the Elastic Stack Server, which hosts the Elastic Stack that consists of docker containers running Elasticsearch: Kibana, Filebeat, and Logstash.

**Figure 3:**
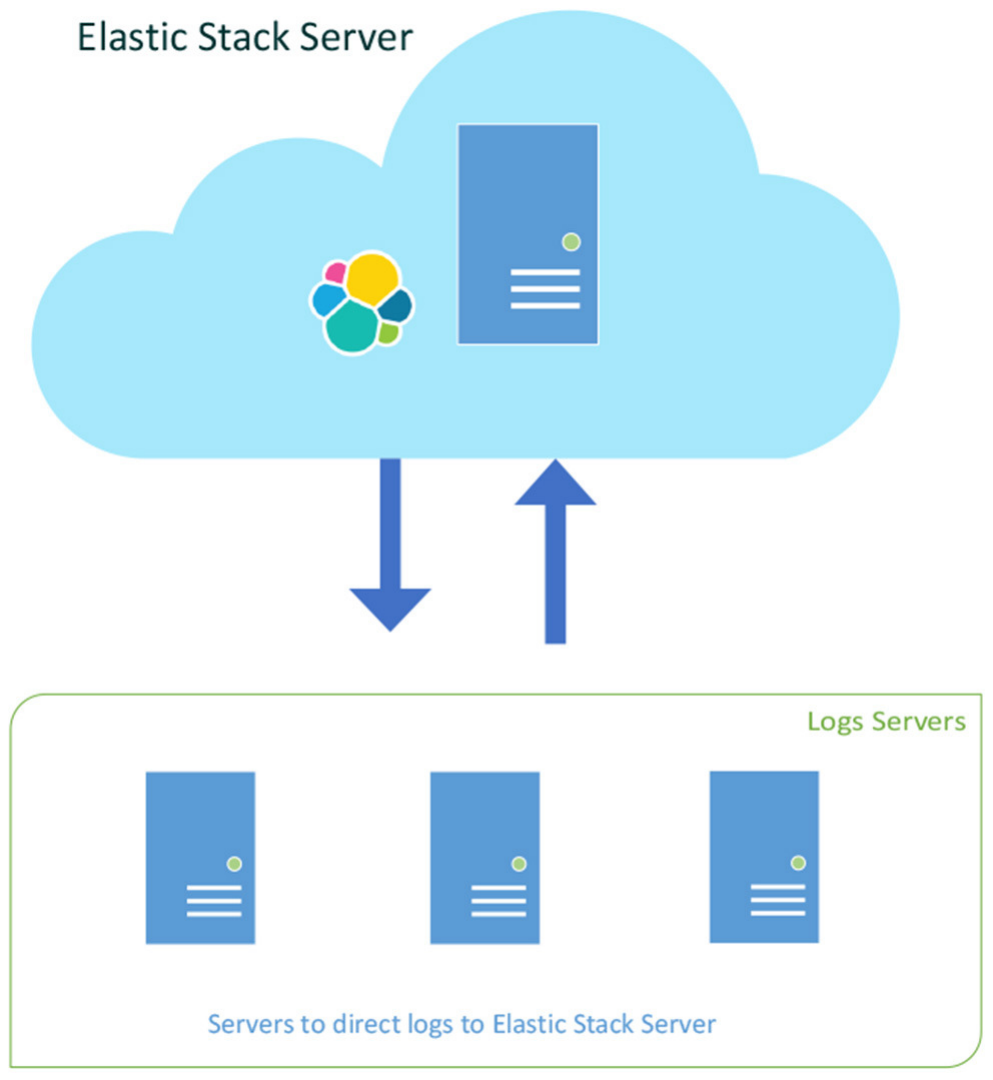
Three log servers sending their logfiles to the Elastic Stack Server.

In addition to the Elastic server, there are three virtual machines that send their logs to the Elastic server. As seen in Figure 3, the Elastic server is placed on a separate network and the docker containers communicate with each other, as well with the other virtual machines.

**Figure 4:**
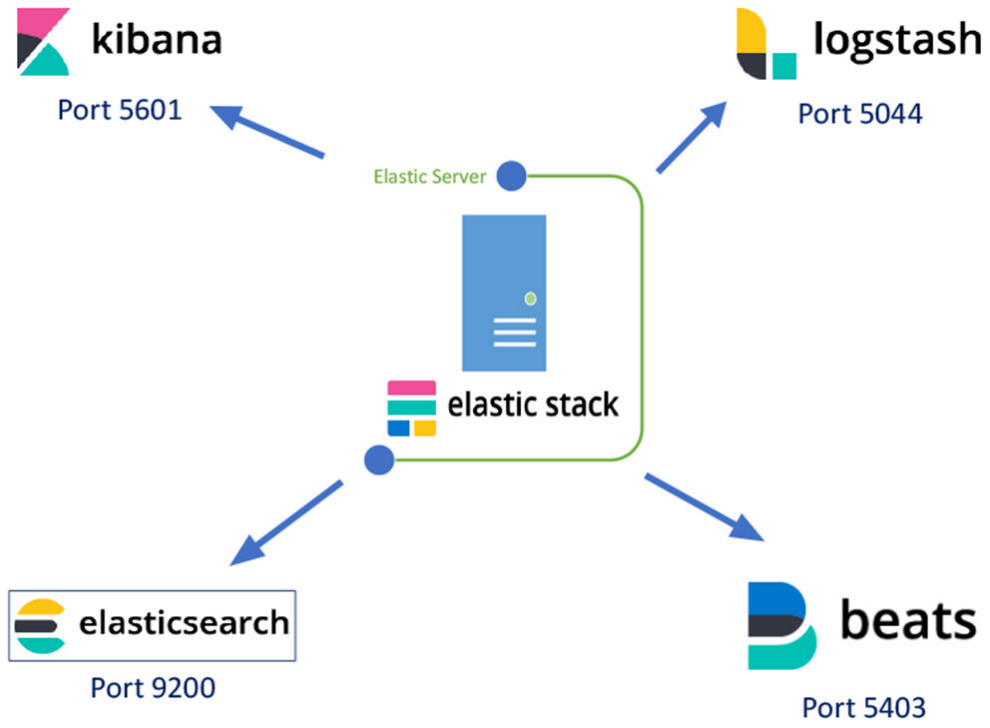
The Elastic Stack applications and their port numbers.

The docker containers of the Elastic Stack use four different TCP ports, as shown in Figure 4 where Elasticsearch uses port 9200, Kibana port 5601, Logstash port 5044 and Beat port 5043.

In addition, the ESRally benchmarking tool is run in a docker image, and the implementation of the algorithm is based on this deployment of docker containers.

The main server that runs the docker containers and the algorithm has the specifications listed in Table 2.

**Table 2:** Elastic stack server specification.

**Operating system**	**Linux Ubuntu16.04 xenial**
CPU	E5530 @ 2.40GHz
RAM	23 Gb
Disk space	439G
Hardware architecture	x86 64
Processor	Intel(R) Xeon(R)

### 3.4. The Document Generator

The document generator is an application written in python that connects to the Elasticsearch cluster and generates fake data. The data generated are in the JSON format, the format used by Elasticsearch, and are referred to as documents. After generating the documents, the code collects the generated data into bulk and sends them to the bulk API of the Elasticsearch node. The API request inserts the generated data into the connected Elasticsearch node. For indexing, the code generates five indexes for five different sets of data.

### 3.5. The Optimizer Algorithm

The optimizer algorithm implements the concept of SPSA (Simultaneous Perturbation Stochastic Approximation) (Spall et al., 1992) in order to tune the configuration of Elasticsearch. The number of parameters to tune is the same for all the experiments. However, the initial values of each parameter will vary. In the algorithm, the initial parameters will have random values, and there will be a variable for each parameter that defines the next step size of that parameter.

Once the initial parameters have been assigned a random value, the algorithm will update the Elasticsearch cluster using an API from Elasticsearch, which allows the settings of a node or a cluster to be updated. The updating task is simply an HTTP request containing information about the parameter that is to be updated and the values.

ESRally produces a JSON file as output. The output is useful for gaining an overview of the current performance of the Elasticsearch node. With the statistics included in the JSON file, we can retrieve the mean throughput of indexing and latency of operations.

Elasticsearch configuration includes several parameters. However, the implemented algorithm uses the four parameters listed in Table 3. A detailed description of the parameters can be found in the official documentation of Elasticsearch.

**Table 3:** The selected elasticsearch parameters.

**Parameter**
translog.sync.interval
indices.recovery.max.bytes.per.sec
index.flush.threshold.size
index.referesh.interval

### 3.6. The SPSA Algorithm

The objective function of the algorithm uses the ESRally output to obtain the indexing throughput and latency time of other operations. The objective function formula for a given configuration θ is given by:


(1) $$\begin{array}{r} f\left(\theta \right)=\frac{Indexing}{Response\;Time} \end{array}$$

where *Indexing* is the mean indexing throughput, i.e., the number of indexed documents, and *ResponseTime* is the mean latency time of operations, i.e., the latency of different search queries types being performed on the Elasticsearch cluster.

The goal of the SPSA algorithm in this article is to maximize the objective function *f*(θ) by updating the configuration parameters θ of the Elasticsearch over time. In order to optimize the configuration we resort to a modified version of the Simultaneous Perturbation Stochastic Approximation algorithm (Kumar et al., 2017). The implemented algorithm in this project is presented in Algorithm 1. It starts with initial parameters θ_0_ = θ, then, for each iteration *n*, the algorithm will generate a perturbation vector Δ_*n*_ which will be used to test a pair of “perturbed” configurations θ_*n*_+Δ_*n*_ and θ_*n*_−Δ_*n*_. Both the performance of configuration θ_*n*_+Δ_*n*_ and that of θ_*n*_−Δ_*n*_ will be evaluated. Let $\theta_{n}^{+}$ denote θ_*n*_+Δ_*n*_ and let $\theta_{n}^{-}$ denote θ_*n*_−Δ_*n*_.

**Table:** **Algorithm 1 :** Modified Simultaneous Perturbation Stochastic Approximation.

1: Initial parameters θ∈ℝ
2: Initial Step Size for each parameter Δ
3: for *n* = 0, 1, 2, …, *N* **do**
4: Generate perturbation vector Δ_*n*_∈ℝ
5: Compute *f*(θ_*n*_+Δ_*n*_)
6: Compute *f*(θ_*n*_−Δ_*n*_)
7: Calculate, *DP*_*n*_, the relative difference between *f*(θ_*n*_+Δ_*n*_) and *f*(θ_*n*_−Δ_*n*_) given by $DP_{n}=2\frac{\left|f\left(\theta_{n-1}+\Delta_{n}\right)-f\left(\theta_{n-1}-\Delta_{n}\right)\right|}{f\left(\theta_{n-1}+\Delta_{n}\right)+f\left(\theta_{n-1}-\Delta_{n}\right)}$
8: Calculate new step size δ_*n*_(*i*) for each parameter *i* as $\delta_{n}\left(i\right)=sign\left(\Delta_{n}\left(i\right)\right)\delta_{min}\left(i\right)\left(1+DP_{n}\right)$
9: Update the value of each parameter *i* for the next iteration *n*+1 following a gradient ascent-like update given by $\theta_{n+1}\left(i\right)=\Pi \left[\theta_{n}\left(i\right)+\delta_{n}\left(i\right)sign\left(f\left(\theta_{n}^{+}\right)-f\left(\theta_{n}^{-}\right)\right)\right]$ where Π is a projection that confines each parameter θ_*n*+1_(*i*) within the minimum and maximum values.
10: end **for**

Depending on the sign of $\left(f\left(\theta_{n}^{+}\right)-f\left(\theta_{n}^{-}\right)\right)$ each parameter value will get either increased or decreased in the same direction, as in a gradient ascent method. In fact, each parameter *i* is updated in the same direction of the “best” perturbated configuration either θ_*n*_+Δ_*n*_ or θ_*n*_−Δ_*n*_. The magnitude of the change to δ_*n*_(*i*) for each parameter *i* is proportional to the magnitude of the relative difference *DP*_*n*_ between *f*(θ_*n*_+Δ_*n*_) and *f*(θ_*n*_−Δ_*n*_), and it is at least δ_*min*_(*i*). The step size is expressed by:


(2) $$\begin{array}{r} \delta_{n}\left(i\right)=\delta_{min}\left(i\right)\left(1+DP\right) \end{array}$$

Thus we use the sign of $f\left(\theta_{n}^{+}\right)-f\left(\theta_{n}^{-}\right)$, as well as the “sign” of the perturbation for each parameter *i* based on Δ_*n*_(*i*) to obtain the direction of update for each parameter. Intuitively, if Δ_*n*_(*i*) is positive, and $f\left(\theta_{n}^{+}\right)>f\left(\theta_{n}^{-}\right)$ then θ_*n*_(*i*) will increase. Similarly, if Δ_*n*_(*i*) is negative, and $f\left(\theta_{n}^{+}\right)>f\left(\theta_{n}^{-}\right)$ then θ_*n*_(*i*) will decrease. To avoid that the value of any parameter *i* gets outside the range [δ_*min*_(*i*), δ_*max*_(*i*)], which is the user defined range, we resort to a projection Π that confines each parameter θ_*n*+1_(*i*) within the minimum and maximum values. The projection can be also expressed using maximum, Max(.,.), and minimum, Min(.,.), operators as:


(3) $$\theta_{n+1}(i)=Min[Max(\delta_{min}(i),\theta_{n}+\delta_{n}(i)sign(f(\theta_{n}^{+})-f(\theta_{n}^{-})),\delta_{max}(i)]$$

### 3.7. Optimizer Data Flow

**Figure 5:**
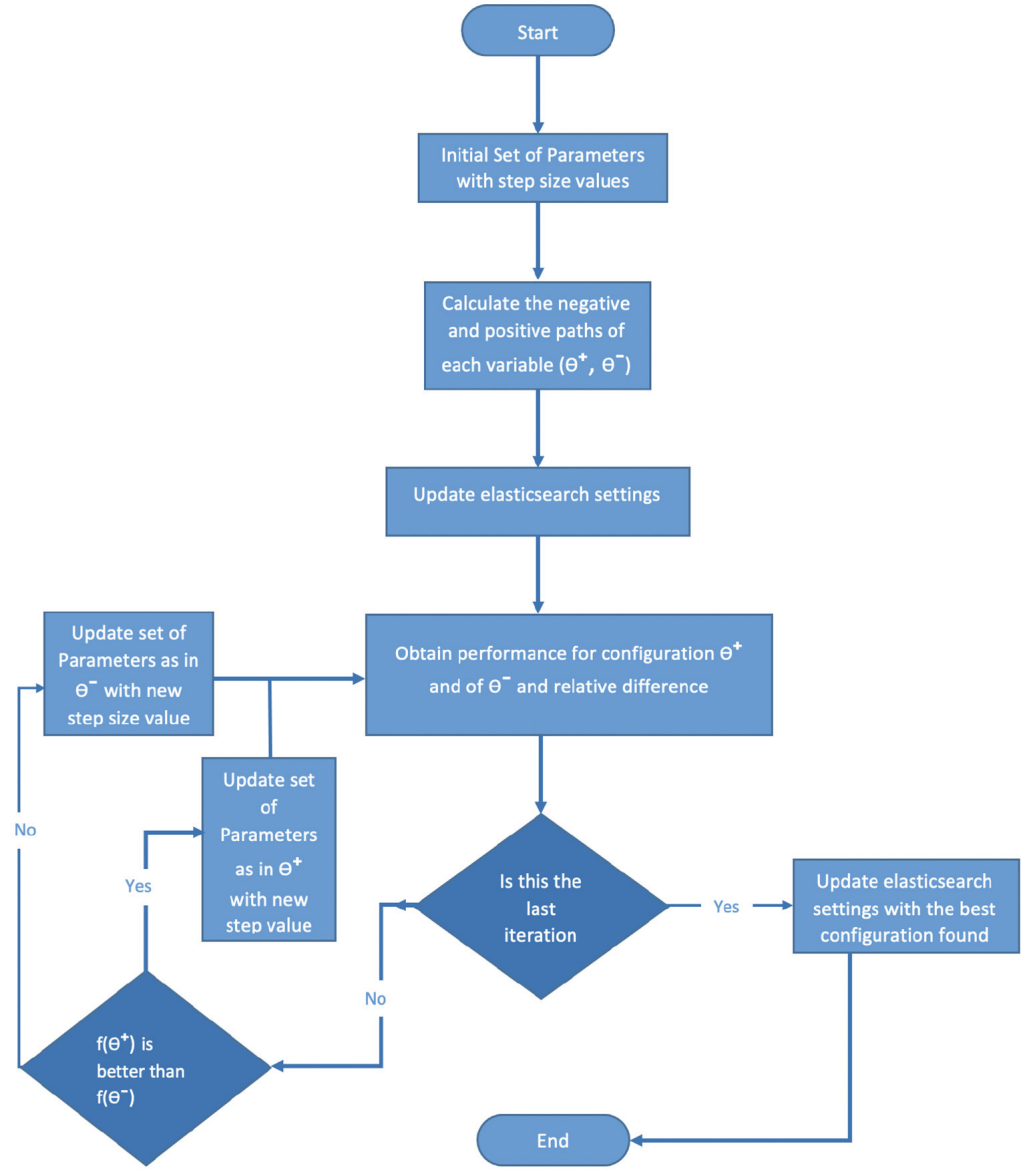
Optimizer algorithm data flow.

The optimizing algorithm can be presented in a data flow diagram, as shown in Figure 5. The data flow diagram starts with *Initial Set of Parameters with Step Size Values*, that is, the parameters that will be tuned by the algorithm. For each parameter, there is a step size that will be updated for each iteration. Then *Calculate the negative and positive paths of each variable*, where θ^+^ presents a set of values to add to each parameter from the selected set of parameters, and θ^−^ are the negative values of these values. After that, *Update Elasticsearch settings* with both θ^+^ and θ^−^, then *Obtain performance for configuration* θ^+^ *and* θ^−^ *and relative difference*, if the iteration is not the last one, then check which objective function value is better than it, θ^+^ or θ^−^; the best value will then be updated in either the stage *Update set of parameters as in* θ^+^ *with new step size value* or the θ^−^ one as seen in the data flow diagram (Figure 5). Then the new set of parameters will be used to update the Elasticsearch settings as before. If the iterations are finished, then update Elasticsearch with the best set of parameters from the previous iterations.

## 4. Results

For each of the tests performed, there is an indexing operation where new data are inserted in the Elasticsearch cluster in order to test the performance of the indexing task. After this, several types of operations will be performed on these indexed data, each with several types of search queries. All of the tests have a list of specifications as shown in the following list:

1. The number of documents: The number of data inserted in Elasticsearch, a document is represented by a JSON object.
2. Bulk Size: How many documents to index per request
3. The number of types of Operations: Performing different types of search queries such as aggregations, range, match all, etc.
4. The number of iterations per operation: keep repeating an operation a specific number of times.

There are three different sets of documents: Taxi rides data, GeoNames Data and HTTP Log Data. All the three data sets used are from the rally-tracks (Mitterdorfer, 2022b) of the Elasticsearch benchmarking tool Rally and are publicly available. As an example of the three types, the GeoNames data represent geographical information about specific areas, and a typical sample is shown in Listing 1. Such data include country code, population, timezone, etc. The total number of data-fields in the GeoNames documents is 11.

**Listing 1:**
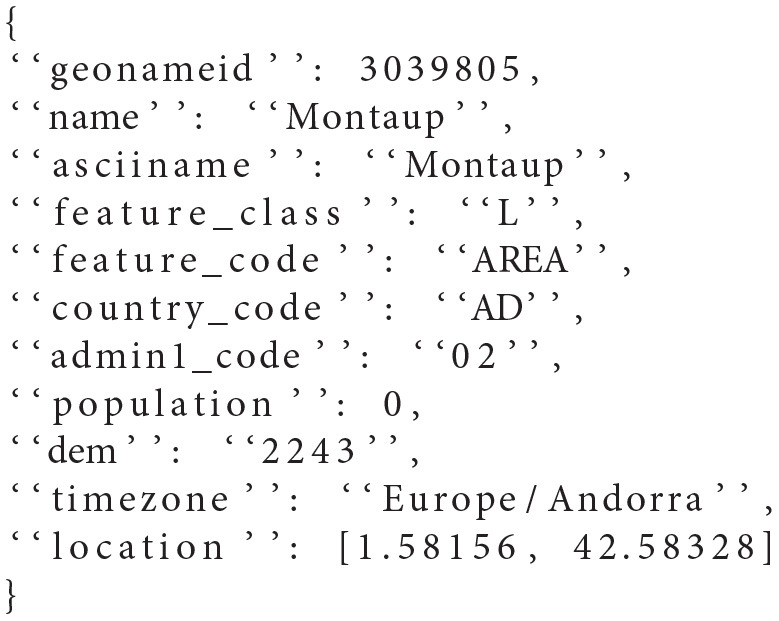
Example of GeoNames Document

In the Table 4, the meta-data for each of the three different sets of data is shown.

**Table 4:** Meta-data for the three sets of documents.

	**Taxi-rides**	**Geo Names**	**HTTP Log**
Bulk size	165,346,692	11,396,505	2,708,746
Types of operations	6	22	10
Data fields	18	11	5

The following subsections present the results of the objective function from running 100 iterations on each of these three data sets. For each iteration, there are two objective function results, and the direction of the tuning will follow the one with the best result.

### 4.1. Taxi Rides Data

**Figure 6:**
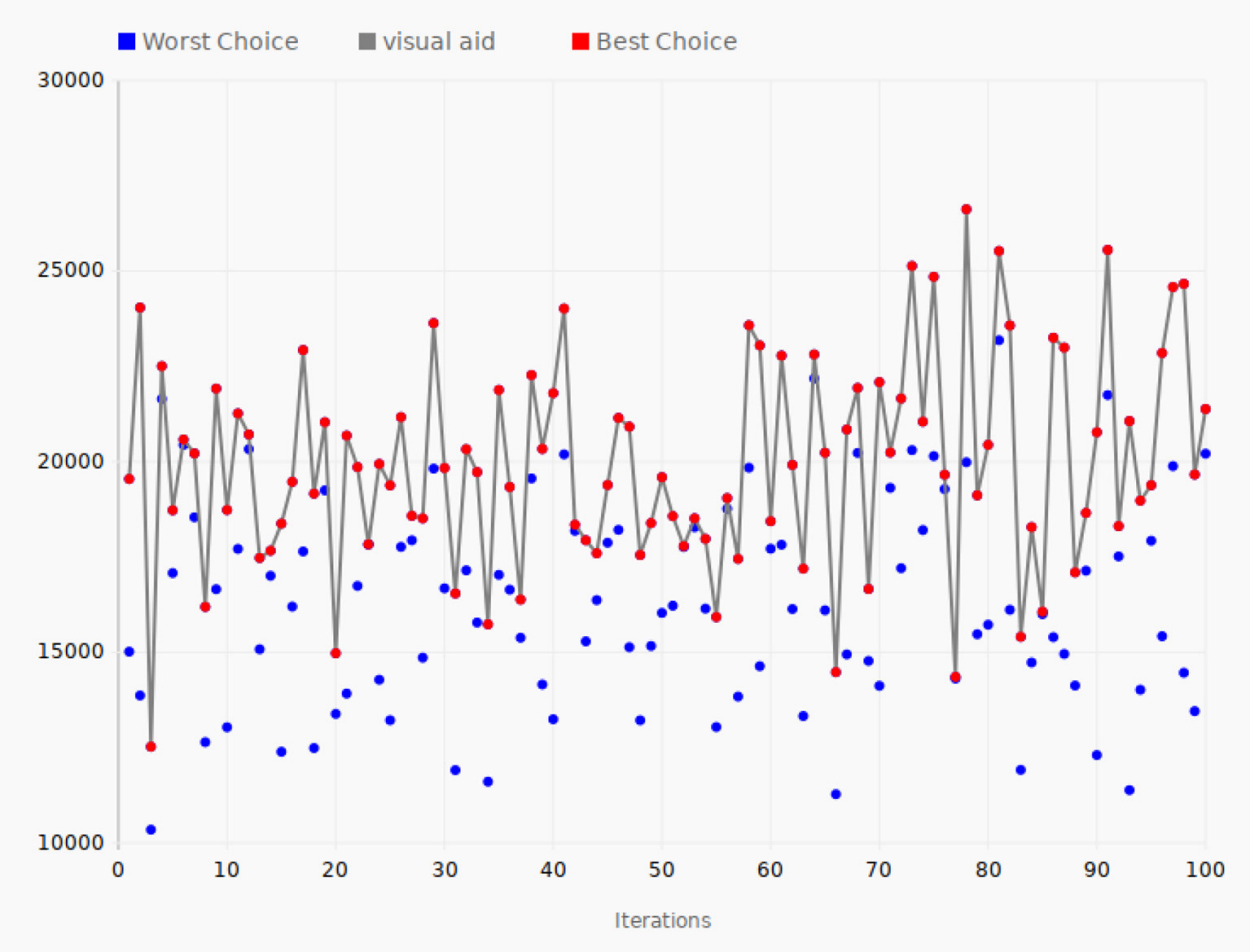
Tuning iterations—taxi rides data. The gray line connects the best choices that give the largest value of the objective function for each of the 100 iterations.

The Taxi Rides documents contain data such as pickup location, pickup dateline, passenger account, improvement surcharge, etc. There are a huge number of documents in this data set, 165 million, and each document contains 18 data fields. Figure 6 shows the tuning performance of the algorithm run using the Taxi Rides data. For each of the iterations, a positive and a negative direction is tested when changing the parameters and the objective function calculated for these two choices. The red line connecting the red dots of the graph shows the best direction for the given iteration, that is, the set of parameters that yields the largest value of the objective function. The path chosen by the SPSA algorithm is thus shown by the red line. The blue dots show the worst direction and hence always represent a lower value. In the first iteration, the objective function value of the best direction was 19,547, while the value for the other direction was 15,018. The set of parameters leading to the largest value was then chosen by the SPSA algorithm, and these parameters were used when considering a new choice between two directions in the next iteration. In iteration 2, the value of the best direction increased to 24,040, yielding the best configuration so far, while the other direction, as shown by the second blue dot, was as low as 13,869. However, in iteration 3, the best value decreased to 12,525, which was the lowest value in all the one hundred iterations of the experiment, except for some of the worst direction values. The goal of the experiment was to move around in the configuration space in this way and seek the best configuration parameters in terms of achieving the highest possible value of the objective function. The largest value occurred in iteration 78, where the objective function reached a peak value of 26,619. This means that the set of parameters used in iteration 78 produced the best performance of all the parameter sets tested in the experiment, making it the optimal choice based on these 100 iterations of the SPSA algorithm.

The 200 data points correspond to the same number of configurations of Elasticsearch. The average value of the objective function for all the 200 data points could be a measure of a typical value you would get if you selected a random configuration of all the parameters, just like for the value of the first iteration. However, the algorithm searches for the configurations that yield the largest value of the objective function, so the average is more likely to be somewhat higher than a completely random choice of parameters. In any case, the optimal value for the objective function found by the algorithm gives an indication of how successful the algorithm is. In the case of taxi rides data, the average value of the objective function for all the data points is 18,751 and the peak value is 26,619, a 42% improvement compared to the average value of all the tested configurations.

### 4.2. GeoNames Data

**Figure 7:**
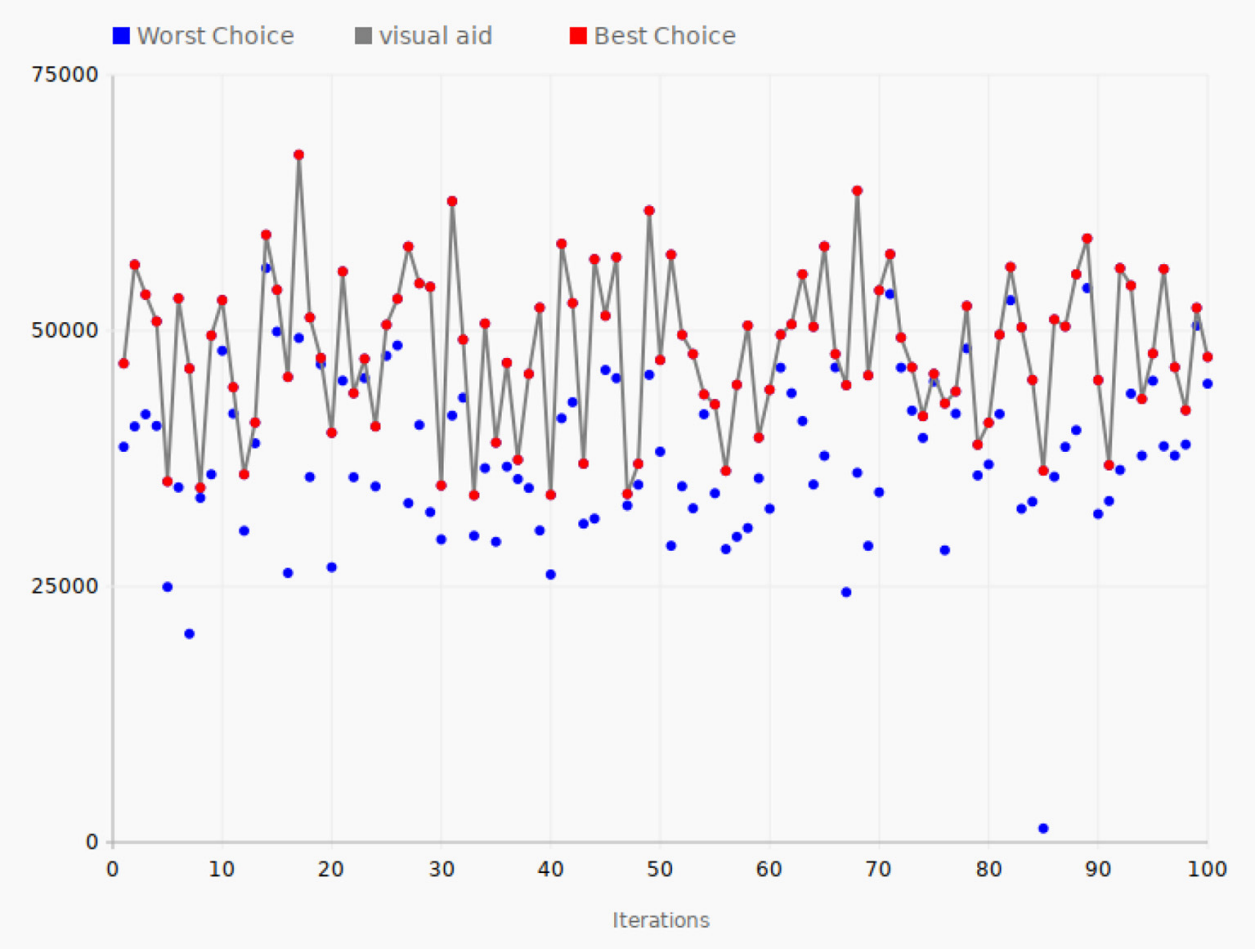
Tuning iterations—GeoNames. The gray line connects the best choices that give the largest value of the objective function for each of the 100 iterations.

A typical document containing geographical data was shown in Listing 1. These data are from the GeoNames database, which is a freely accessible database of geographical data. Figure 7 shows the tuning performance of 100 iterations of the SPSA algorithm applied to documents containing GeoNames data.

As in Figure 6, the red line shows the path in the configuration space of the selected best direction of the algorithm, while the blue dots show the other direction leading to a lower value for the objective function.

The starting value of the objective function was 46,800, while it ended with a value of 47,432. The lowest performance was in iteration 33, with the value of 33,920. On the other hand, in iterations 31, 68, and 49, the values of the objective function reached the large values of 62,659, 63,690, and 61,740. The highest value reached was 67,200 in iteration 17, the best performance of the system during the test.

In the case of GeoNames data, the average value of the objective function for all the 200 data points is 44,759 and the peak value found by the SPSA algorithm is 67,200, which is a 50% improvement compared to the average value of all the tested configurations.

### 4.3. HTTP Log Data

**Figure 8:**
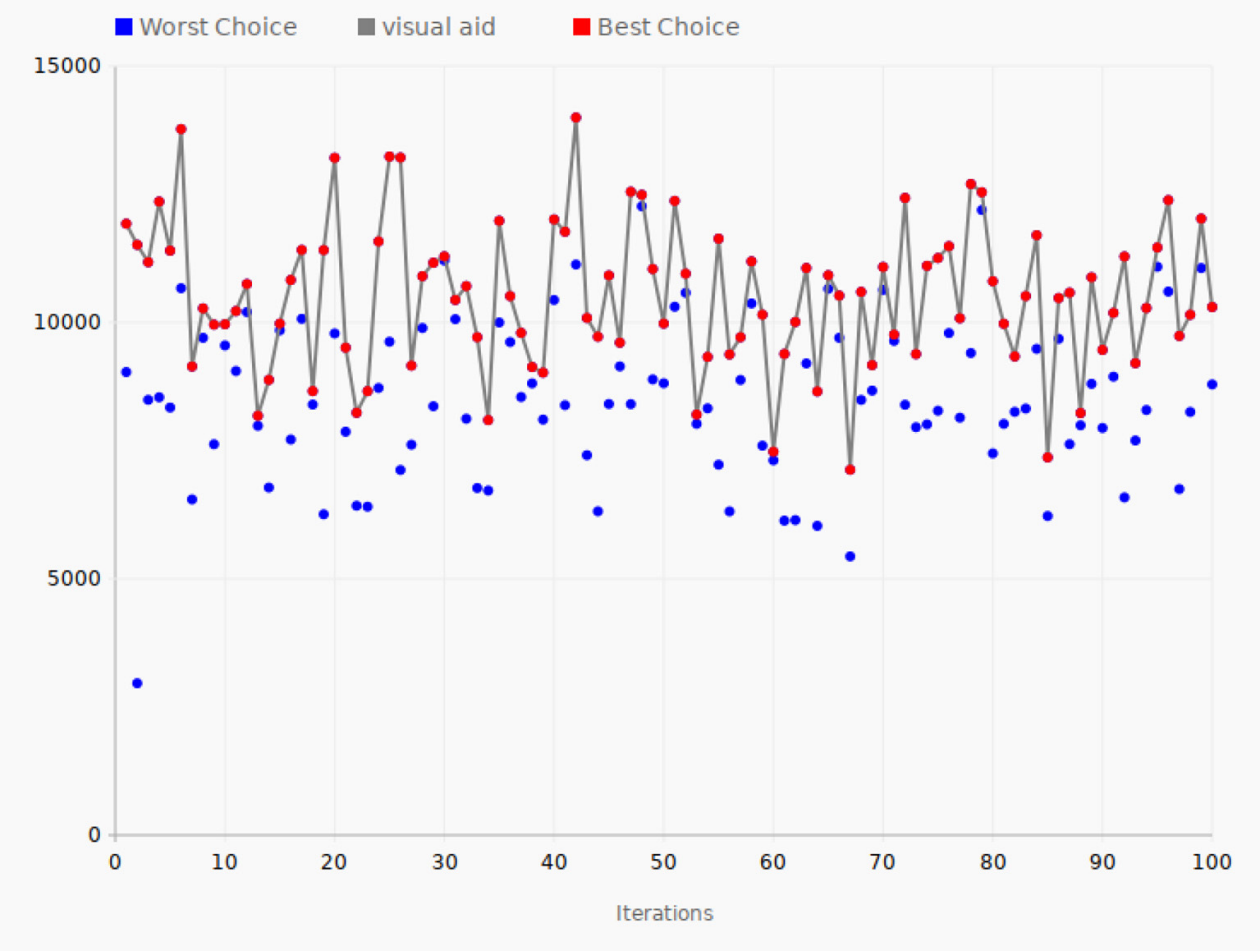
Tuning process—HTTP logs data. The gray line connects the best choices that give the largest value of the objective function for each of the 100 iterations.

Figure 8 shows the tuning performance of the algorithm applied to HTTP logs. In the first iteration, the objective function value was 11,926. It then increased to 12,358 in iteration 4, which was the second-largest value in this test. The tuning process reached its best value in iteration 42 with the value 13,996. The lowest value, 7,126, was recorded at iteration 67. Finally, in the last iteration, the objective function value was 10,300, which was approximately the average of the best values. The set of parameter values in iteration 42 is the best set in terms of high performance in comparison with all the other iterations.

In the case of HTTP data, the average value of the objective function for all the 200 data points is 9853 while the peak value found by the SPSA algorithm is 13,996, a 42% improvement compared to the average value for all the tested configurations, as in the case of the taxi rides data.

## 5. Discussion and Conclusion

The goal of this article was to create an SPSA algorithm that would automate parameter tuning of Elasticsearch and improve its performance. We were able to change the Elasticsearch configuration dynamically without resetting the node, thereby enabling us to continuously search for the best parameter setting while at the same time carrying out the work.

In the implemented algorithm, the SPSA algorithm iterates several times to test the current performance of the system. In each iteration, the algorithm observes the system by applying a set of parameters, which are a representation of parameter combination values. These values are updated for every iteration, and the updates are dependent on the previous iteration in which the system performance was observed.

It is difficult to find the correct set of parameters manually in order to tune the performance when the Elasticsearch node stores a lot of data and responds to many queries. Therefore, using optimization solutions such as our SPSA algorithm improves the process of choosing the set of parameters that will result in good performance.

Performing several experiments on the designed system resulted in efficient tuning of the Elasticsearh parameters. Overall, the Elasticsearch configuration and the implemented algorithm yielded the best combined values for low latency of operations and a high number of inserted documents. The actual experiments showed that, in the three cases with different document types, the algorithm resulted in an improvement in the objective function of between 42 and 50 percent compared to what could be expected if the parameters had not been tuned.

The implemented SPSA algorithm ensures dynamic tuning of the Elasticsearch nodes. It keeps performing tests on the node while observing its performance. The best solution is saved, and once all tests are done, the Elasticsearch node is updated with the best solution without any need to reset the node. The authors of the article introducing the SPSA algorithm claims that it is extremely useful in cases when the dimensionality is high and the observations are costly (Kumar et al., 2016). The experimental results of this work confirms that the SPSA algorithm is efficient for a different but similar case where the number of configuration parameters is a bit smaller.

In general, configuring Elasticsearch has to be done manually by specifying which parameters need to be changed to achive better performance. This is impractical when data are continuously inserted. The patterns of the incoming data might change at any time and this could also lead to a change of the optimal parameter configuration. The proposed solution ensures automatic reconfiguration by continously testing different sets of parameters and updating the configuration with the best solution.

## Data Availability Statement

The raw data supporting the conclusions of this article will be made available by the authors, without undue reservation.

## Author Contributions

HH, MS, and AY contributed to the conception and design of the study. MS organized and performed the experiments under the supervision of HH and AY. HH wrote the first draft of the manuscript. All authors contributed to manuscript revision, read, and approved the submitted version.

## Conflict of Interest

The authors declare that the research was conducted in the absence of any commercial or financial relationships that could be construed as a potential conflict of interest.

## Publisher's Note

All claims expressed in this article are solely those of the authors and do not necessarily represent those of their affiliated organizations, or those of the publisher, the editors and the reviewers. Any product that may be evaluated in this article, or claim that may be made by its manufacturer, is not guaranteed or endorsed by the publisher.
